# The 2020 race towards SARS-CoV-2 specific vaccines

**DOI:** 10.7150/thno.53691

**Published:** 2021-01-01

**Authors:** Tomasz M. Karpiński, Marcin Ożarowski, Agnieszka Seremak-Mrozikiewicz, Hubert Wolski, Donald Wlodkowic

**Affiliations:** 1 Chair and Department of Medical Microbiology, Poznań University of Medical Sciences, Wieniawskiego 3, 61-712 Poznań, Poland.; 2 Department of Biotechnology, Institute of Natural Fibres and Medicinal Plants, Poznań, Poland.; 3 Division of Perinatology and Women's Disease, Poznań University of Medical Sciences, Poznań, Poland.; 4 Laboratory of Molecular Biology in Division of Perinatology and Women's Diseases, Poznań University of Medical Sciences, Poznań, Poland.; 5 Department of Pharmacology and Phytochemistry, Institute of Natural Fibres and Medicinal Plants, Poznań, Poland.; 6 Division of Obstetrics and Gynecology, Tytus Chałubiński's Hospital, Zakopane, Poland.; 7 School of Science, RMIT University, Melbourne, VIC, Australia.

**Keywords:** severe acute respiratory syndrome coronavirus 2, SARS-CoV-2, coronavirus disease 2019, COVID-19, vaccine, clinical trials

## Abstract

The global outbreak of a novel severe acute respiratory syndrome coronavirus 2 (SARS-CoV-2) highlighted a requirement for two pronged clinical interventions such as development of effective vaccines and acute therapeutic options for medium-to-severe stages of “coronavirus disease 2019" (COVID-19). Effective vaccines, if successfully developed, have been emphasized to become the most effective strategy in the global fight against the COVID-19 pandemic. Basic research advances in biotechnology and genetic engineering have already provided excellent progress and groundbreaking new discoveries in the field of the coronavirus biology and its epidemiology. In particular, for the vaccine development the advances in characterization of a capsid structure and identification of its antigens that can become targets for new vaccines. The development of the experimental vaccines requires a plethora of molecular techniques as well as strict compliance with safety procedures. The research and clinical data integrity, cross-validation of the results, and appropriated studies from the perspective of efficacy and potently side effects have recently become a hotly discussed topic.

In this review, we present an update on latest advances and progress in an ongoing race to develop 52 different vaccines against SARS-CoV-2. Our analysis is focused on registered clinical trials (current as of November 04, 2020) that fulfill the international safety and efficacy criteria in the vaccine development. The requirements as well as benefits and risks of diverse types of SARS-CoV-2 vaccines are discussed including those containing whole-virus and live-attenuated vaccines, subunit vaccines, mRNA vaccines, DNA vaccines, live vector vaccines, and also plant-based vaccine formulation containing coronavirus-like particle (VLP). The challenges associated with the vaccine development as well as its distribution, safety and long-term effectiveness have also been highlighted and discussed.

## Introduction

In December 2019, the first cases of human infections by the zoonotic SARS-CoV-2 (initially named 2019-nCoV) were reported. Those initially occurred in Wuhan (China) and centered on a population of wet market traders where live seafood, as well as live farmed and wild animals, were commonly sold 1. The virus causes a respiratory disease named as coronavirus disease 2019 (COVID-19) 2. The number of infected patients has continued to increase at an alarming rate. In comparison from December 31, 2019, up to August 25, 2020, 23,673,902 cases of COVID-19 have been reported worldwide. More than two months later, up to November 04, 2020, there were double the number of infected patients, with 47,594,234 cases of COVID-19 and 1 215 892 deaths. On November 04, 2020, the highest numbers of patients with COVID-19 was stated in the United States (9,383,979), India (8,313,876), Brazil (5,566,049) and Russia (1,673,686) 3.

Similar to historical experience with majority of respiratory track infectious diseases, compliance with hygiene guidelines can only slow down the overall number of infections due to restriction of exposure pathways. An effective vaccine, if successfully developed, has been broadly emphasized to become the most effective strategy in the global fight against COVID-19 pandemic.

In this review, we present a critical update on latest advances and progress in an ongoing race to develop 51 different vaccines against SARS-CoV-2. The motivation of our work is to provide an up-to-date analysis focused on registered clinical trials (current as of November 04, 2020) that fulfill the international safety and efficacy criteria in the vaccine development. The data were obtained from six clinical trial registries of the European Union 4, India 5, China 6, Australia and New Zealand 7, Clinical Trials of US National Library of Medicine 8, Pan African Clinical Trials Registry 9, Cuban Registry of Clinical Trials 10, Indonesia Registry 11, and ISRCTN 12.

We discuss the requirements as well as benefits and risks of diverse types of SARS-CoV-2 vaccines are discussed including those containing whole-virus and live-attenuated vaccines, subunit vaccines, mRNA vaccines, DNA vaccines, live vector vaccines, and also plant-based vaccine formulation containing coronavirus-like particle (VLP). The challenges associated with the vaccine development as well as its distribution, safety and long-term effectiveness have also been highlighted and discussed.

## SARS-CoV-2 biology

Coronaviruses name comes from a characteristic image that resembles a solar corona when observed using a transmission electron microscope 13. Today over seven human coronaviruses have been identified. Four of them characterized by relatively low pathogenicity are HCoV-229E, HCoV-OC43, HCoV-NL63, and HCoV-HKU1. They are responsible for seasonal and usually mild infections of the upper respiratory tract. These four coronaviruses are responsible for up to 15-30% of common colds in adults 14. The remaining are the highly pathogenic SARS-CoV-1, MERS-CoV, and SARS-CoV-2 viruses, responsible for severe lower respiratory tract infections. Human coronaviruses are now classified into the kingdom Riboviria, order Nidovirales, family Coronaviridae and the subfamily Orthocoronavirinae 15. Four genera are distinguished in this subfamily: Alphacoronavirus, Betacoronavirus, Deltacoronavirus, and Gammacoronavirus. Viruses 229E and NL63 belong to Alphacoronaviruses, while OC43, HKU1, SARS-CoV-1, MERS-CoV, and SARS-CoV-2 belong to Betacoronaviruses. Alpha- and Beta-coronaviruses can cause infections in various species of mammals, including humans, but population of bats is their natural habitat. Wild birds are a common reservoir of delta- and gamma-coronaviruses. One of the most dangerous features of coronaviruses is their capability to break through the species barrier. In this regard, a plethora of reports demonstrated transfers from wild to domestic birds and some species of mammals, including even marine mammals 16,17.

SARS-CoV-2 has single-stranded, non-segmented RNA of positive polarity (+ ssRNA). RNA can act directly as mRNA in protein translation 15. SARS-CoV-2 belongs to a new evolutionary branch within the genus Betacoronavirus and has 79% genetic similarity with SARS-CoV and nearly 50% similarity with MERS-CoV 18. Phylogenetic analyses showed that SARS-CoV-2 is closely related (88% similarity) to two coronaviruses occurring in bats, namely bat-SL-CoVZC45 and bat-SL-CoVZXC21. Bats are likely the natural hosts for SARS-CoV-2, and pangolins can be one of the intermediate hosts significant to viral transfer to humans 19,20. SARS-CoV-2 can be transmitted directly from person to person. Main ways are close contact and emission of respiratory droplets by infected person during coughs or sneezes. The virus can also be transmitted indirectly by touching contaminated surfaces or objects and touching the face, eyes, or mouth 21.

## Antigens of SARS-COV-2

The antigens of the virus include all of its elements, including proteins, lipids, polysaccharide, and nucleic acids. The whole-cell antigens are used for developing the whole virus killed and live-attenuated vaccines 22.

**Figure 1:**
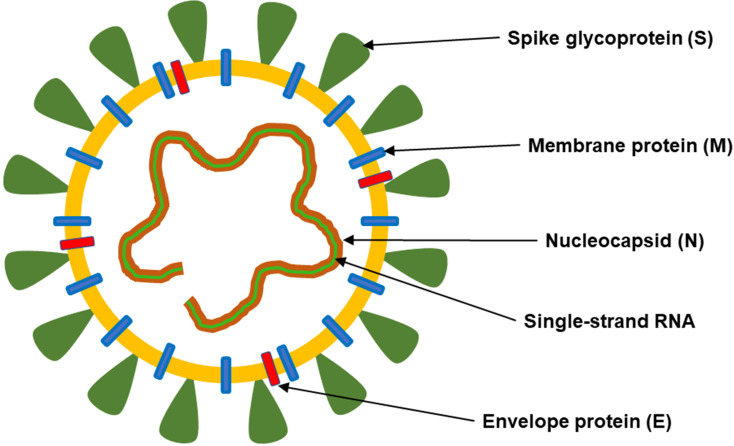
**The structure of SARS-CoV-2.** This coronavirus is an enveloped, positive-sense RNA virus and contains four main structural proteins, including spike (S), membrane (M), envelope (E) and nucleocapsid (N) proteins.

Major structural proteins: spike protein (S), nucleocapsid protein (N), membrane glycoprotein (M), and envelope protein (E) can be distinguished in the SARS-CoV-2 structure (**Figure 1**).

### Spike glycoprotein (S)

The S protein is surface fusion glycoprotein, which can be directly recognized by the host immune system. It gives the virion a corona or crown-like appearance 23. Protein S mediates the entry of the virus into host cells. The mechanisms of SARS-CoV-2 infection rely on the entry of the virus into human cells by angiotensin-converting enzyme 2 (ACE2) receptor 2,24. The virus attacks mainly epithelial cells in the respiratory and gastrointestinal tracts 25. Binding capacity to the ACE2 receptor is approximately 10-20 times stronger in the case of SARS-CoV-2 than the SARS-CoV. Simultaneously, the density of the ACE2 is higher in adults than in children 26. This can potentially explain why adults have a higher predisposition to infection and severe disease in comparison to children.

The S protein forms a trimer on the viral membrane and consists of two subunits (S1 and S2) 23. The S1 subunit contains two domains such as the N-terminal domain (NTD) and the C-terminal domain (CTD). The latter includes the receptor-binding domain (RBD). RBD is responsible for host cell receptor binding. The S2 subunit facilitates membrane fusion between the viral and host cell membranes 27. The S protein binds to the angiotensin-converting enzyme 2 (ACE2) receptor and directly facilitates viral entry 25. It is studied for use as antigens in vaccines both as full-length S protein, and also as the RBD domain, the S1 subunit, and NTD 22. The S protein is one of the essential immunodominant proteins of coronaviruses, inducing host immune responses 28. This glycoprotein is the most often target for SARS-CoV-2 developed vaccines, including adenoviral, RNA-based, DNA-based, and protein subunit vaccines 29.

### Nucleocapsid protein (N)

The N protein has multiple functions. Self-association of the N protein is required for the packaging the viral genome and formation of the viral capsid. It is also involved in virus budding, RNA replication, and mRNA transcription 30. The N protein can inhibit the activity of cyclin-cyclin-dependent kinase (cyclin-CDK) complex, which leads to inhibition of S phase (genome replication) progression in the mammalian cell cycle 31. The N protein of SARS-CoV can activate an AP-1 pathway, which regulates many cellular processes, including cell proliferation, differentiation, and apoptosis 32. Nucleocapsid protein can also inhibit type I interferon (IFN) and immune responses and simultaneously activate cyclooxygenase-2 (COX-2) leading to inflammation in the lungs 33.

### Membrane glycoprotein (M)

The M protein is a transmembrane glycoprotein and has three transmembrane domains. It binds to the nucleocapsid and is involved in virus assembly 34. It interacts with the N protein to encapsulate the RNA genome and generate virions 35. The M protein can inhibit production of COX-2 and activation of nuclear factor kappa B (NFκB), thus enhancing the viral proliferation 33. In the SARS virus, this protein has been reported to induce apoptosis by through the canonical caspases cascade 36.

### Envelope protein (E)

The E protein is a small membrane protein with reported ion channel activity 37. This protein also plays a role in viral assembly, virion release, and viral pathogenesis 34. This protein was reported to have viroporin activity, and can mediate pathogenic processes and induce cytotoxicity 33. In contrast to other major structural proteins, the E protein is not suitable for use as an immunogen 22. The E protein affects the production of pro-inflammatory cytokines IL-1, IL-6 and TNF 38.

## Types of SARS-CoV-2 vaccines

The vaccine against SARS-CoV-2 should meet at least the following requirements: minimize undesired immunopotentiation, be suitable for adult healthcare workers, be suitable for adults > 60 years old or with underlying diabetes or hypertension, and be suitable for long-term stockpiling 39.

**Figure 2:**
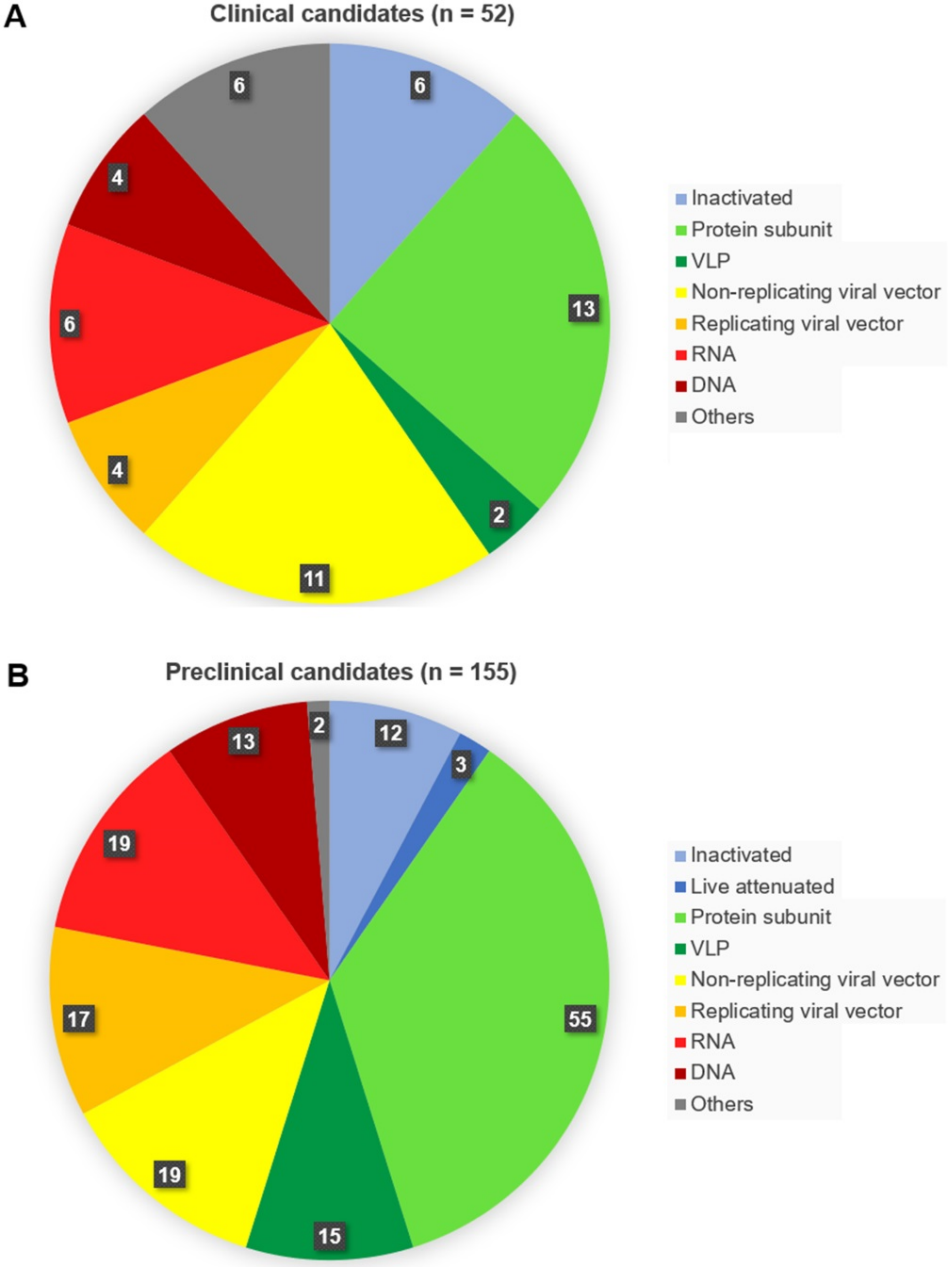
** Number of vaccine candidates against SARS-CoV-2 in (A) clinical and (B) preclinical development, selected according to vaccine platform technology.** Classical (conventional) platforms include the inactivated virus, live attenuated virus, protein subunit, and virus-like particle vaccines. To the next-generation platforms belong viral vectored, RNA, DNA and antigen-presenting cells vaccines.

**Figure 3:**
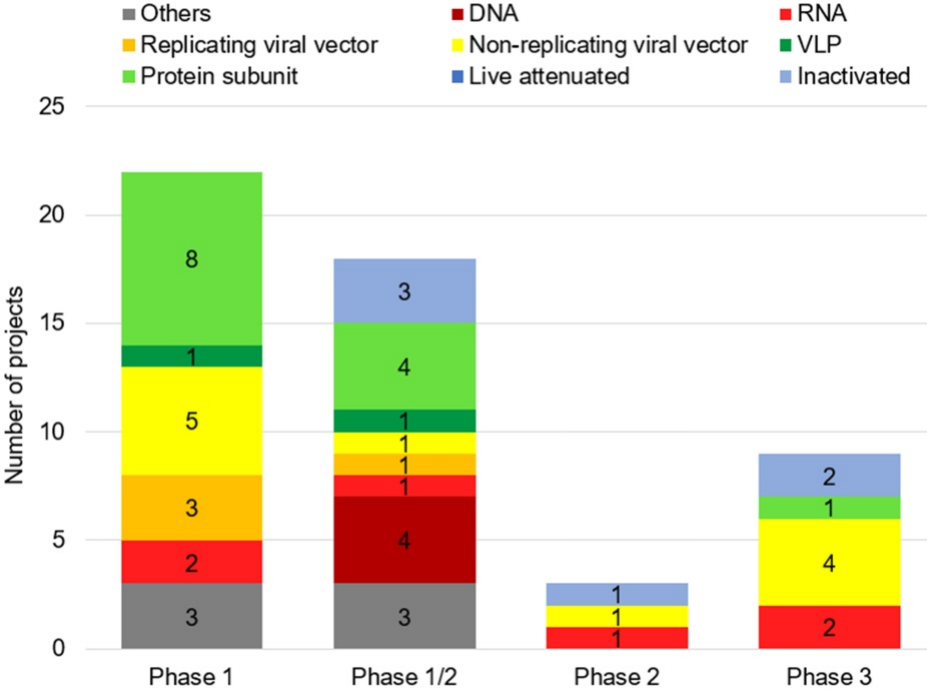
**Number of candidates of SARS-CoV-2 vaccines by phase of clinical trial.** As of November 04, 2020, fifty-two vaccines against SARS-CoV-2 are undergoing clinical trials, including 22 in Phase 1, 18 in Phase 1/2, three in Phase 2, and 9 in Phase 3.

As of November 04, 2020, fifty-two vaccines against SARS-CoV-2 are undergoing clinical trials, including 9 in Phase 3, three in Phase 2, 18 in Phase 1/2 and 22 in Phase 1 (**Table 1**). These include 13 protein subunit candidates, 11 non-replicating viral vector vaccines, 6 inactivated, 6 RNA and 4 DNA vaccines, four replicating viral vectored, two virus-like protein (VLP), and 6 others (**Figures 2A,B & Figure 3**). In Phase 1 are vaccines in small-scale safety, given to a small group of volunteers, in Phase 2 are vaccines in expanded safety, given to hundreds of volunteers, in Phase 3 are vaccines in large-scale safety and efficacy, given to thousands of volunteers 40.

Simultaneously, according to the data obtained from the World Health Organization database up to 155 candidate vaccines are recently in preclinical tests 41. Among these are 55 protein subunit candidates, 19 RNA, 19 non-replicating viral vector vaccines, 17 replicating viral vector vaccines, 15 VLP, 13 DNA candidates, 12 inactivated, 3 containing live attenuated virus, and two others (**Figure 2A & B**).

**Figure 4:**
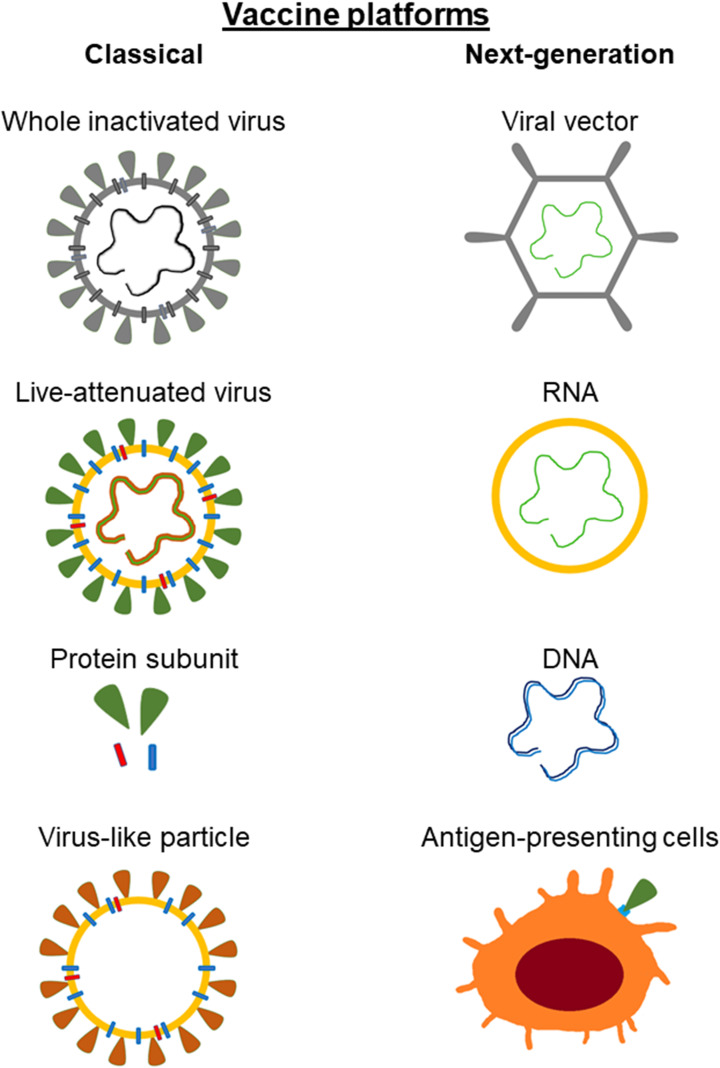
**Classical and next-generation vaccine platforms used in development against SARS-CoV-2.** Classical (conventional) include those that are based on vaccines already licensed and used in humans. The next generation vaccines do not require the actual viral particle and can be developed solely on the sequence of the antigenic viral proteins.

Currently, both classic vaccine platforms and next-generation vaccine platforms can be distinguished (**Figure 4**). Classic (conventional) include those that are based on vaccines already licensed and used in humans. These vaccines are either virus-based or protein-based. Virus-based vaccines may contain live attenuated virus or inactivated virus. Vaccines contained live attenuated viruses are created by multiple passages of the virus in cell culture, leading to its loss of virulence. This type of vaccines leads to the development of a mild infection. Vaccines based on the inactivated virus ensure that the virion cannot replicate and is not contagious. However they do require adjuvants to properly stimulate the immune response. Protein-based vaccines may consist of a virus-purified protein, a recombinant protein, or virus-like particles (VLP). Inactivated virus vaccines and protein-based vaccines require adjuvants to stimulate the immune system 42-45. Virus-like particles contain the structural proteins of viral capsid. Simultaneously, they do not have the viral genome and critical non-structural proteins 46.

The next generation vaccines do not require the actual viral particle and can be developed solely on the sequence of the antigenic viral proteins. The material present in the vaccine containing information about the protein coding sequence leads to its biosynthesis and thus to an immune response. Next-generation vaccines include viral vector, nucleic acid-based and antigen-presenting cells vaccines 43,44,46. Characteristics of selected vaccines are presented in **Table 2.**

## Classical vaccine platforms

### Inactivated Vaccines

Inactivated vaccines belong to the classic strategy for viral vaccinations. These vaccines contain multiple antigenic components and therefore, can potentially induce diverse immunologic responses 22. In comparison to live-attenuated vaccines, they reportedly have less reactogenicity and are typically characterized by weaker immune responses. Inactivated vaccines may require multiple inoculations and strong adjuvants to be effective. The advantages are, however, that development process is well established and streamlined but requires handling of the live virus 47. At present the most advanced candidates are in phase 3 of the clinical trials, including the Sinovac vaccine (Sinovac Research and Development Co., Ltd./Butantan Institute), and the COVID-19 vaccine produced on Vero cells (Wuhan Institute of Biological Products Co., Ltd./Sinopharm/Beijing Institute of Biological Products Co., Ltd.). One candidate is in Phase 2 (Shenzhen Kangtai Biological Products Co., Ltd.; Beijing Minhai Biotechnology Co., Ltd.). Three other candidates are still in phase 1/2 of the clinical trials such as BBV152 (Bharat Biotech International Limited), QazCovid-in (Research Institute for Biological Safety Problems) and SARS-CoV-2 vaccine from the Chinese Academy of Medical Sciences. In preclinical evaluation are 12 inactivated candidates.

### Live-Attenuated Vaccines

Live-attenuated vaccines also belong to the classic strategy for viral vaccinations and contain multiple antigenic components 22. The advantage of whole-virus vaccines is strong immunogenicity and stimulation of Toll-like receptors. The protection is usually long-lasting as shown by examples of vaccines against smallpox, poliovirus and measles. Their effectiveness is confirmed, among others by eradication of smallpox and very close to successful eradication of polio diseases 48. The development process is well established but also requires handling of live virus particles during production 47. In immunocompromised persons, live vaccines can theoretically lead to development of fully-fledged infection. Therefore, they must have additional tests to confirm their safety 49. At present there are no live-attenuated vaccines in clinical stages. Three live-attenuated vaccines are in preclinical evaluation, developed by Mehmet Ali Aydinlar University, Codagenix/Serum Institute of India and Indian Immunologicals Ltd/Griffith University 41.

### Subunit Vaccines

Subunit vaccines contain one or more antigens with strong immunogenicity. They are safer and less difficult to manufacture because they do not require handling of live virus particles during the production process. Their disadvantage is that this type of vaccine often requires effective adjuvants to obtain stronger immune responses 22. In general, subunit vaccines consisted of immunogenic proteins, which have properties of immunoprotective antigens. They are safer than immunization with all inactivated pathogen 50. The first group of the SARS-CoV-2 subunit vaccines uses the S protein as antigens. Examples of such vaccines are SCB-2019 vaccine (Clover Biopharmaceuticals AUS Pty Ltd.), NVX-CoV2373 (Novavax), Covax-19 (GeneCure Biotechnologies; Vaxine Pty Ltd.), the vaccine developed by University of Queensland and MVC-COV1901 (Medigen Vaccine Biologics Corp.). The second group are subunit vaccines employing the RBD domain of S protein as antigen: KBP-COVID-19 (Kentucky BioProcessing, Inc.) and vaccine from Anhui Zhifei Longcom Biologic Pharmacy Co., Ltd. Vaccine NVX-CoV2373 (Novavax) is currently in phase 3, and others subunit vaccines against SARS-CoV-2 are in phase 1 or 2 of clinical trials. In preclinical evaluation are 55 protein subunit candidates.

In studies of NVX-CoV2373 vaccine, 83 and 25 participants received the vaccine with adjuvant and without adjuvant, respectively. In most persons, local and systemic reactogenicity was absent or mild. The most often adverse effects reported were headache, fatigue, and malaise. There have been no presented severe adverse events. In adjuvant-based trials enhanced immune responses and induction of T helper 1 (Th1) cells was demonstrated. Moreover, two doses of adjuvant vaccine induced production of anti-spike IgG with neutralization responses. Anti-spike IgG levels were approximately 100 times higher than in patients with symptomatic Covid-19 51.

### Virus-like particles vaccines (VLP)

VLP vaccines contain many viral structural proteins and mimic the organization and conformation of viruses. These vaccines do not contain the viral genome and therefore are non-infectious and very safe. The use of a combination of structural proteins from different viruses produces recombinant VLPs 52,53.

Of particular interest are vaccines produced in plants. The plant cells are ideal platforms for the production of oral delivery vaccines. They are also commonly referred to as edible vaccines 54. The genes for viral proteins are most commonly introduced to host plant using pathogenic bacteria *Agrobacterium*. After infection, the gene of interest incorporates into nuclear or chloroplast genome. This transformation leads to biosynthesis of very large amounts of virus-like particles in the plant host 55. It was shown that plant vaccines manufactured using *Agrobacterium* infiltration-based transient expression platform (*Nicotiana benthamiana*) against avian influenza H5 (AIV) pandemic and influenza A viruses (A/H1N1, A/H3N2) were safe and well-tolerated. Moreover, it was confirmed as inducing strong and cross-reactive humoral and cellular responses 56. This type of vaccine was shown a promising source of the vaccine of Newcastle disease 57, of Lyme disease 58, a glycoprotein of bovine viral diarrhea virus 59, and recombinant colicin M 60. Among candidates against SARS-CoV-2 are also RBD SARS-CoV-2 HBsAg VLP vaccine in Phase 1/2 (Serum Institute of India), and plant-derived coronavirus-VLP vaccine (Medicago), that recently entered phase 1 trials. In preclinical evaluation are 15 VLP candidates.

## Next-generation vaccine platforms

### Viral Vector-Based Vaccines

Live vector-based vaccines are live recombinant viruses, which deliver vaccine genes or antigens to the target host tissues. Attenuation is essential for safety; however, despite this vector-based vaccines have an elevated risk of certain adverse events (**Table 2**). The viral vector imitates infection caused by genuine virus. Therefore it can induce stronger cellular immune response than recombinant protein vaccine 47. There is currently a number of non-replicating vaccines in development based on both adenoviral and lentiviral vectors. The adenoviral-vectored includes being in Phase 3 ChAdOx1 nCoV-19/AZD1222 (University of Oxford/Astra Zeneca; University of Witwatersrand, South Africa), Ad26.COV2.S (Janssen Vaccines & Prevention B.V.), Ad5-nCoV (Insitute of Biotechnology, Academy of Military Medical Sciences, PLA of China/CanSino Biologics Inc.) and Gam-COVID-Vac (Gamaleya Research Institute of Epidemiology and Microbiology, Health Ministry of the Russian Federation). Moreover, 2019-nCOV candidate (Institute of Biotechnology, Academy of Military Medical Sciences, PLA of China) is in Phase 2, and three vaccines: VXA-CoV2-1 (Vaxart), hAd5-S-Fusion+N-ETSD (ImmunityBio, Inc.) and defective Simian adenovirus vectored GRAd-COV2 (ReiThera Srl) are in Phase 1. Lentiviral vector-based vaccines in development include currently the LV-SMENP-DC in Phase 1/2 and Pathogen-specific aAPC vaccine in Phase 1, both tested by Shenzhen Geno-Immune Medical Institute.

In Phase 1/2 of clinical trials is replicating VSV vectored rVSV-SARS-CoV-2-S/IIBR-100 vaccine (Israel Institute for Biological Research), and in Phase 1 are also four replicating viral vectored vaccines (Table 1), it is intranasal flu vectored DelNS1-2019-nCoV-RBD-OPT1 (Beijing Wantai Biological Pharmacy), measles-vector based TMV-083 (Institut Pasteur/Themis Bioscience/Coalition for Epidemic Preparedness Innovations), VSV-vector based V590 (Merck Sharp & Dohme Corp.), and modified vaccinia virus Ankara vectored MVA-SARS-2-S (Universitätsklinikum Hamburg-Eppendorf). In preclinical evaluation are currently 19 non-replicating and 17 replicating candidates.

In a preliminary report of a phase 1/2, a single-blind, randomized controlled trial the ChAdOx1 nCoV-19 was shown an acceptable safety profile. Authors did not observe any serious adverse effects related to ChAdOx1 nCoV-19. Spike-specific T-cell responses peaked on day 14 post inoculation. Neutralizing antibody responses against SARS-CoV-2 were also detected in more than 90% of participants 61.

Good tolerability and immunogenicity were also reported for recombinant adenovirus type-5 (Ad5) vectored vaccine, currently in phase 1 trial. Among 108 participants neutralizing antibodies increased significantly on day 14 and peaked 28 days post-vaccination. After administration of a single dose of the vaccine, a four-fold increase in the number of RBD-binding antibodies was found in up to 100% of participants, and of neutralizing antibodies in up to 75% of participants. However, most of the individuals exhibited adverse side effects such as pain at the injection site, fever, fatigue, headache, and muscle pain. No serious and systemic side effects were reported so far 62. In the phase 2 trial, two doses of the Ad5-vectored vaccine induced significant neutralizing antibody responses to live coronavirus. Severe adverse reactions were reported in about 10% of participants. No serious adverse effects were documented 63.

In Russian studies it was shown that Gam-COVID-Vac vaccine was safe and well tolerated by 76 participants. The adverse events were mild and most common were pain at injection site, fever, headache, asthenia, and muscle and joint pain. After 21 days, in all subjects production of antibodies against SARS-CoV-2 glycoprotein was observed. Formation of T-helper (CD4^+^) and T-killer (CD8^+^) cells was confirmed in all participants at day 28 64.

### mRNA Vaccines

mRNA vaccines are characterized by high potency what is connected with adjuvant. They have short production cycles and potential for rapid and low-cost manufacturing 65. These vaccines induce activation of both B cell responses and T cell cytotoxicity 66. In some mRNA vaccines, lipid nanoparticles (LNPs) are used to enhance the mRNA transfection efficacy 67. One vaccine mRNA-1273 (Moderna TX, Inc./National Institute of Allergy and Infectious Diseases) is currently in Phase 3 of clinical trials and one BNT162 (BioNTech/Fosun Pharma/Pfizer) in Phase 2/3. CVnCoV (CureVac AG) is in Phase 2 and ARCT-021 (Arcturus Therapeutics, Inc.) in Phase 1/2. The other two vaccines, LNP-nCoVsaRNA (Imperial College London) and vaccine from Yunnan Walvax Biotechnology Co., Ltd, are currently in Phase 1 of clinical trials. In preclinical evaluation are 19 RNA candidates.

The mRNA-1273 vaccine consists of a mRNA drug surrounded by lipid nanoparticles (LNPs). It was recently observed that immunization with mRNA-1273 vaccine induced strong SARS-CoV-2 neutralizing activity in non-human primates 68. In phase 1 trials, it has been also demonstrated that the mRNA-1273 vaccine was effective in eliciting immune responses in all tested 45 participants when used in two injections 28 days apart. Side effects included predominantly pain and inflammation at the injection site, fatigue, chills, headache, and myalgia. Systemic adverse effects after the first vaccination were reported as mild to moderate, but were more severe and frequent after the second vaccination 69.

### DNA Vaccines

DNA vaccines mainly contain plasmid DNA that encodes one or more antigens. They need to enter the nucleus for the production of antigen proteins to take place 70. This type of vaccine eliminates the need for using live viruses and are amenable for freeze-drying and long-term storage 47. So far, four SARS-CoV-2 DNA vaccines are under development including INO-4800 (International Vaccine Institute; Inovio Pharmaceuticals), nCov vaccine (Cadila Healthcare Ltd.), AG0301-COVID19 (AnGes, Inc.) and GX-19 (Genexine, Inc.). All of these candidates are in Phase 1/2 of clinical trials. In preclinical evaluation are currently 13 DNA candidate vaccines.

## Challenges with accelerated vaccine development

It has been shown that candidate vaccines lead to production of antibodies against SARS-CoV-2 51,61,62. However, it is unknown how long immunity will last after vaccination. Antibody titers in persons after SARS-CoV-1 or MERS-CoV infections often weakened after 1-3 years 71,72. Studies in rhesus macaques showed that anti-S-IgG antibodies did not inhibit SARS-CoV infection, but instead exacerbated macrophage-mediated lung damage 73. In persons with COVID-19, it was shown that antibodies can be undetectable three months after infection 74. Today we know, that both neutralizing antibodies and cellular immune responses are important in responding to SARS-CoV-2, and potential vaccines should elicit both of these responses 75,76. Some case reports presented that in patients is possible reinfection with SARS-CoV-2 77,78. Unfortunately, in some persons, second infection can be harder than first, which may indicate a short time of persistence of antibodies after primary infection. It is also possible, as in Denga virus, occurrence of antibody-dependent enhancement (ADE) leading to exacerbation of COVID-19 by anti-SARS-CoV-2 antibodies 79.

According to Logunov et al., the COVID-19 vector vaccine candidates were well tolerated and did not cause serious adverse events during phase 1 and 2 studies 64. However, according to observation during other trials 61-63, after vaccination against SARS-CoV-2 may occur side effects including injection site pain, fever, fatigue, headache, muscle pain, diarrhoea, nausea, appetite impaired, swelling, and cough. At present, phase 3 studies are underway for the Moderna and BioNTech/Pfizer vaccines, the Oxford/AstraZeneca viral vector vaccine and the Johnson & Johnson viral vector vaccine, enrolling 30,000-40,000 subjects 80. However, at last scientists urge caution in global vaccine race because the next phase of clinical trials is hold after “suspected adverse event” (the inflammation of spinal cord) in a person who received the vaccine in the UK 80,81.

The main purpose of vaccination is to eliminate or significantly reduce disease transmission in a population by inducing herd immunity. The herd immunity in healthy persons is obtained in case of influenza after vaccination of above 80% of population 82, and in case of measles more than 90% 83. It is estimated that 67% of the population are required to be vaccinated to obtain herd immunity to SARS-CoV-2 84. This would require production of over 5 billion doses of one-dose vaccine or more than 10 billion of double-doses vaccine. The challenge will be also production and distribution of vials to store the vaccines and syringes to administer them.

Until outbreak of current pandemic, the development of vaccines and their approval lasted on average more than 15 years 85. Vaccines against SARS-CoV-1 have not been introduced for 17 years since the outbreak of the epidemic and MERS-CoV for 6 years. Therefore, in recent pandemic the timing of the first vaccine implementation is unknown, but due to social and economic costs the pressure for rapid development and implementation is immense. There are advantages and disadvantages to each vaccine platforms. No DNA or RNA vaccine is currently licensed and approved for therapeutic use in humans. Viral vector vaccines exhibit potential risk such as chromosomal integration and oncogenesis, and generally cannot be used in immunocompromised subjects. Protein vaccines need adjuvants to enhance immunological response. At the same time global scale deployment of attenuated vaccines is always associated with risks of pathogen reactivation and regaining its virulence in the future 86. At present every vaccine technology represents a compromise between good efficacy, safety, and a lack of side-effects.

## Future outlook

Development and dissemination of vaccines has been one of the great achievements of contemporary medicine. However, despite benefits, their clinical use carries also some risks. It should be remembered that e.g. the influenza virus changes its antigenic structure. Within each type and subtype of seasonal influenza virus, new major antigenic variants arise approximately every 3-8 years 87. Even if we manage to develop and deploy a COVID-19 vaccine the main uncertainty lies in how mutations of the SARS-CoV-2 strains will affect its effectiveness. According to Mercatelli and Giorgi 88, the SARS-CoV-2 virus has at least six identified strains and its variability is not high. This means that the vaccines under development can be potentially effective against major strains of the virus 88.

Global efforts are continuing to attempt to inhibit and eradicate of the COVID-19 pandemic. The unprecedented race towards effective and suitable candidates for SARS-CoV-2 vaccines is ongoing. The most advanced so far are eight very different vaccines that just entered phase 3 trials. The results obtained in clinical trials so far are promising, but more research is required on the efficacy and safety of these vaccines. However, the development and introduction of a vaccine for use is a multi-stage and long-term process. In addition, the production and distribution of a huge number of vaccine doses will be a problem. Ultimately, even if a vaccine becomes available, it will take many years to get herd immunity in human society.

## Figures and Tables

**Table 1:** Clinical trials of SARS-CoV-2 vaccines, current as of October 19, 2020

Name of vaccine	Type of vaccine	Stage of clinical trial	Identifier	Developer/manufacturer
Sinovac	Inactivated	Phase 3	NCT04582344	Sinovac Research and Development Co., Ltd./Butantan Institute/Health Institutes of Turkey.
		Phase 3	NCT04456595	
		Phase 3	INA-WXFM0YX	
		Phase 1/2	NCT04551547	
		Phase 1/2	NCT04352608	
		Phase 1/2	NCT04383574	
COVID-19 vaccine (Vero cells)	Inactivated	Phase 3	ChiCTR2000039000	Wuhan Institute of Biological Products Co., Ltd./Sinopharm,
		Phase 3	ChiCTR2000034780	
		Phase 1/2	ChiCTR2000031809	
		Phase 3	NCT04560881	Beijing Institute of Biological Products Co., Ltd.
		Phase 1/2	ChiCTR2000032459	
NVX-CoV2373/SARS-CoV-2 rS/Matrix-M1 Adjuvant	Recombinant S protein subunit with matrix-M adjuvant	Phase 3	EudraCT 2020-004123-16	Novavax
		Phase 3	NCT04611802	
		Phase 2	NCT04533399	
		Phase 1	NCT04368988	
mRNA-1273	Lipid nanoparticle (LNP)-encapsulated mRNA	Phase 3	NCT04470427	Moderna TX, Inc./National Institute of Allergy and Infectious Diseases.
		Phase 2	NCT04405076	
		Phase 1	NCT04283461	
ChAdOx1 nCoV-19/AZD1222	Non-replicating adenovirus vectored	Phase 3	NCT04540393	University of Oxford/Astra Zeneca/Serum Institute of India
		Phase 3	NCT04516746	
		Phase 3	ISRCTN89951424	
		Phase 2/3	CTRI/2020/08/027170	
		Phase 2/3	NCT04400838	
		Phase 2/3	EudraCT 2020-001228-32	
		Phase 1/2	NCT04324606	
		Phase 1/2	EudraCT 2020-001072-15	
		Phase 1/2	PACTR202006922165132	
		Phase 1/2	NCT04444674	University of Witwatersrand, South Africa
Ad26.COV2.S	Non-replicating adenovirus 26 vectored	Phase 3	NCT04505722	Janssen Vaccines & Prevention B.V.
		Phase 1/2	NCT04436276	
Ad5-nCoV	Non-replicating adenovirus type 5 vectored	Phase 3	NCT04540419	Insitute of Biotechnology, Academy of Military Medical Sciences, PLA of China/CanSino Biologics Inc.
		Phase 3	NCT04526990	
		Phase 2	NCT04566770	
		Phase 2	ChiCTR2000031781	
		Phase 2	NCT04341389	
		Phase 1/2	NCT04398147	
		Phase 1	ChiCTR2000030906	
		Phase 1	NCT04552366	
		Phase 1	NCT04313127	
Gam-COVID-Vac	Non-replicating adenovirus 5 and 26 vectored	Phase 3	NCT04564716	Gamaleya Research Institute of Epidemiology and Microbiology, Health Ministry of the Russian Federation.
		Phase 3	NCT04530396	
		Phase 2	NCT04587219	
		Phase 1/2	NCT04436471	
		Phase 1/2	NCT04437875	
BNT162a, BNT162b	Nucleoside-modified mRNA	Phase 2/3	NCT04368728	BioNTech/Fosun Pharma/Pfizer
		Phase 1/2	NCT04537949	
		Phase 1/2	EudraCT 2020-001038-36	
		Phase 1/2	NCT04380701	
		Phase 1	ChiCTR2000034825	
SARS-CoV-2 Vaccine (Vero Cells)	Inactivated	Phase 2	ChiCTR2000039462	Shenzhen Kangtai Biological Products Co., Ltd./Beijing Minhai Biotechnology Co., Ltd.
		Phase 1	ChiCTR2000038804	
2019-nCOV vaccine	Non-replicating adenovirus vectored	Phase 2	ChiCTR2000031781	Insitute of Biotechnology, Academy of Military Medical Sciences, PLA of China
		Phase 1	ChiCTR2000030906	
Vaccine (CHO cell)	Recombinant RBD protein subunit	Phase 2	NCT04466085	Anhui Zhifei Longcom Biologic Pharmacy Co., Ltd.
		Phase 1/2	NCT04550351	
		Phase 1	NCT04445194	
CVnCoV	mRNA	Phase 2	NCT04515147	CureVac AG
		Phase 1	NCT04449276	
INO-4800	DNA plasmid	Phase 1/2	NCT04447781	Inovio Pharmaceuticals/International Vaccine Institute
		Phase 1	NCT04336410	
nCov vaccine	DNA plasmid	Phase 1/2	CTRI/2020/07/026352	Cadila Healthcare Ltd.
GX-19	DNA	Phase 1/2	NCT04445389	Genexine, Inc.
AG0301-COVID19	DNA plasmid	Phase 1/2	NCT04527081	AnGes, Inc.
		Phase 1/2	NCT04463472	
BBV152	Whole virion inactivated	Phase 1/2	CTRI/2020/09/027674	Bharat Biotech International Ltd.
		Phase 1/2	CTRI/2020/07/026300	
		Phase 1/2	NCT04471519	
no name	Inactivated	Phase 1/2	NCT04470609	Chinese Academy of Medical Sciences
		Phase 1/2	NCT04412538	
QazCovid-in	Inactivated	Phase 1/2	NCT04530357	Research Institute for Biological Safety Problems
V-SARS	Pill vaccine from heat-inactivated plasma of donors with COVID-19	Phase 1/2	NCT04380532	Immunitor LLC
AV-COVID-19	Autologous dendritic cells loaded with antigens from SARS-CoV-2	Phase 1/2	NCT04386252	Aivita Biomedical, Inc.
EpiVacCorona	Protein subunit	Phase 1/2	NCT04527575	Federal Budgetary Research Institution State Research Center of Virology and Biotechnology “Vector”.
no name	Protein subunit	Phase 1/2	NCT04537208	Sanofi Pasteur/GlaxoSmithKline
KBP-COVID-19	RBD protein subunit	Phase 1/2	NCT04473690	Kentucky BioProcessing, Inc.
FINLAY-FR-1	RBD protein subunit	Phase 1/2	IFV/COR/04	Finlay Vaccine Institute
RBD SARS-CoV-2 HBsAg VLP	VLP	Phase 1/2	ACTRN12620000817943	Serum Institute of India
ARCT-021	Lipid nanoparticle (LNP)-mRNA	Phase 1/2	NCT04480957	Arcturus Therapeutics, Inc.
AlloStim	Bioengineered allogeneic cellular vaccine derived from healthy blood donors	Phase 1/2	NCT04441047	Immunovative Therapies, Ltd.
LV-SMENP-DC	Minigenes engineered based on multiple viral genes, lentiviral vectored (NHP/TYF)	Phase 1/2	NCT04276896	Shenzhen Geno-Immune Medical Institute
rVSV-SARS-CoV-2-S/IIBR-100	Replicating viral VSV vectored	Phase 1/2	NCT04608305	Israel Institute for Biological Research
Pathogen-specific aAPC	Minigenes engineered based on multiple viral genes, lentiviral vectored (NHP/TYF)	Phase 1	NCT04299724	Shenzhen Geno-Immune Medical Institute
GRAd-COV2	Non-replicating defective Simian adenovirus vectored	Phase 1	NCT04528641	ReiThera Srl
hAd5-S-Fusion+N-ETSD	Non-replicating adenovirus Ad5 vectored	Phase 1	NCT04591717	ImmunityBio, Inc.
VXA-CoV2-1	Non-replicating adenovirus Ad5 vectored	Phase 1	NCT04563702	Vaxart
MVA-SARS-2-S	Non-replicating modified vaccinia virus Ankara vectored	Phase 1	NCT04569383	Universitätsklinikum Hamburg-Eppendorf
DelNS1-2019-nCoV-RBD-OPT1	Replicating intranasal based-RBD flu vectored	Phase 1	ChiCTR2000037782	Beijing Wantai Biological Pharmacy
TMV-083	Replicating measles-vector based	Phase 1	NCT04497298	Institut Pasteur/Themis Bioscience/Coalition for Epidemic Preparedness Innovations
V590	Replicating VSV-vector based	Phase 1	NCT04569786	Merck Sharp & Dohme Corp.
no name	mRNA	Phase 1	ChiCTR2000039212	Yunnan Walvax Biotechnology Co., Ltd.
		Phase 1	ChiCTR2000034112	
LNP-nCoVsaRNA	Lipid nanoparticle (LNP)-RNA	Phase 1	ISRCTN17072692	Imperial College London
no name	Recombinant chimeric DC vaccine	Phase 1	ChiCTR2000030750	Shenzhen Third People's Hospital
pVAC	Protein subunit	Phase 1	NCT04546841	University Hospital Tuebingen
UB-612	RBD protein subunit	Phase 1	NCT04545749	United Biomedical Inc./COVAXX
Recombinant vaccine (Sf9)	RBD protein subunit	Phase 1	ChiCTR2000037518	West China Hospital, Sichuan University
SCB-2019	Trimeric spike protein subunit	Phase 1	NCT04405908	Clover Biopharmaceuticals AUS Pty Ltd
Covax-19	Recombinant spike protein subunit with Advax-SM adjuvant	Phase 1	NCT04428073	GeneCure Biotechnologies
		Phase 1	NCT04453852	Vaxine Pty Ltd
no name	Spike protein subunit with MF59 adjuvant	Phase 1	ISRCTN51232965	University of Queensland
			ACTRN12620000674932	
MVC-COV1901	S protein subunit	Phase 1	NCT04487210	Medigen Vaccine Biologics Corp.
no name	Plant-derived coronavirus-like particle (VLP)	Phase 1	NCT04450004	Medicago
bacTRL-Spike	Live *Bifidobacterium longum*, engineered to deliver DNA plasmids	Phase 1	NCT04334980	Symvivo Corporation
no name	Nucleocapsid-GM-CSF protein lactated Ringer's injection	Early Phase 1	NCT03305341, NCT03348670	Han Xu, Sponsor-Investigator, IRB Chair, Medicine Invention Design, Inc.

Data obtained from clinical trial registries of the European Union 4, India 5, China 6, Australia and New Zealand 7, Clinical Trials of US National Library of Medicine 8, Pan African Clinical Trials Registry 9, Cuban Registry of Clinical Trials 10, Indonesia Registry 11, and ISRCTN 12.

**Table 2:** Some characteristics of selected candidate vaccines against SARS-CoV-2 86,89-91

Type of vaccine	Advantages	Disadvantages	Used examples against other pathogens
Inactivated	Easy to prepare; proven technology; safety; multivalent; no adjuvants required; induce strong immune responses	Potential inappropriate for persons with immunosuppression; complicated to scale up manufacturing	Polio
Live-Attenuated	Rapid development; proven technology; multivalent; no adjuvants required; induce strong immune responses	Possibility of reversion; risk for infection; complicated to scale up manufacturing	Measles, Mumps, Rubella, Chickenpox
Subunit	Safety; consistent production; induce strong cellular and humoral immune responses	High cost; lower immunogenicity; require repeated doses and adjuvants; complicated to scale up manufacturing	Pertussis, Hepatitis B, Influenza
Viral Vector-Based	Safety; induces strong cellular and humoral responses	Potential risk for infection, chromosomal integration and oncogenesis; possibly present pre-existing immunity against the vector; risk for inflammatory adverse reactions	Ebola
RNA	Safety; rapid development and production; no risk of genetic integration; possibility of multivalency; induce strong immune responses, both humoral and cell-mediated	Unstable under physiological conditions; possibility of inflammatory reactions; risk for adverse reactions; high cost	Not currently licensed
DNA	Safety; rapid development and production; possibility of multivalency; immune response, both humoral and cell-mediated; long-term stability; possibility of oral formulation	Poor immune responses in humans; repeated doses may cause toxicity; potential risk of genetic integation	Not currently licensed

## References

[1] Wang C, Horby PW, Hayden FG, Gao GF (2020). A novel coronavirus outbreak of global health concern. Lancet.

[2] Zeidler A, Karpiński TM (2020). What do we know about SARS-CoV-2 virus and COVID-19 disease?. J Pre-Clin Clin Res.

[3] COVID-19 situation update worldwide, as of 7 August 2020. European Centre for Disease Prevention and Control. Available at:.

[4] EU Clinical Trials Register - Update. Available at:.

[5] Clinical Trials Registry - India (CTRI). Available at:.

[6] Chinese Clinical Trial Register (ChiCTR) - The world health organization international clinical trials registered organization registered platform. Available at:.

[7] ANZCTR. Available at:.

[8] Home - ClinicalTrials.gov. Available at:.

[9] Pan African Clinical Trials Registry. Available at:.

[10] Welcome to the Cuban Registry of Clinical Trials | Registro Público Cubano de Ensayos Clínicos. Available at:.

[11] Welcome Indonesia Registry Center. Available at:.

[12] ISRCTN Registry. Available at:.

[13] Pancer KW (2018). Pandemiczne koronawirusy człowieka - charakterystyka oraz porównanie wybranych właściwości HCoV-SARS i HCoV-MERS. Post Mikrobiol.

[14] Liu DX, Liang JQ, Fung TS (2020). Human Coronavirus-229E, -OC43, -NL63, and -HKU1. Ref Module Life Sci.

[15] Zeidler A, Karpiński TM (2020). SARS-CoV, MERS-CoV, SARS-CoV-2 comparison of three emerging Coronaviruses. Jundishapur J Microbiol.

[16] Jackwood MW, Hall D, Handel A (2012). Molecular evolution and emergence of avian gammacoronaviruses. Infect Genet Evol.

[17] Paim FC, Bowman AS, Miller L, Feehan BJ, Marthaler D, Saif LJ, Vlasova AN (2019). Epidemiology of Deltacoronaviruses (δ-CoV) and Gammacoronaviruses (γ-CoV) in wild birds in the United States. Viruses.

[18] Lu R, Zhao X, Li J, Niu P, Yang B, Wu H (2020). Genomic characterisation and epidemiology of 2019 novel coronavirus: implications for virus origins and receptor binding. Lancet.

[19] Machhi J, Herskovitz J, Senan AM, Dutta D, Nath B, Oleynikov MD (2020). The natural history, pathobiology, and clinical manifestations of SARS-CoV-2 infections. J Neuroimmune Pharmacol.

[20] Haq EU, Yu J, Guo J (2020). Frontiers in the COVID-19 vaccines development. Exp Hematol Oncol.

[21] Yadav M (2020). Understanding the epidemiology of COVID-19. Eur J Biol Res.

[22] Zhang J, Zeng H, Gu J, Li H, Zheng L, Zou Q (2020). Progress and prospects on vaccine development against SARS-CoV-2. Vaccines.

[23] Millet JK, Whittaker GR (2018). Physiological and molecular triggers for SARS-CoV membrane fusion and entry into host cells. Virology.

[24] Datta PK, Liu F, Fischer T, Rappaport J, Qin X (2020). SARS-CoV-2 pandemic and research gaps: understanding SARS-CoV-2 interaction with the ACE2 receptor and implications for therapy. Theranostics.

[25] Zhang H, Penninger JM, Li Y, Zhong N, Slutsky AS (2020). Angiotensin-converting enzyme 2 (ACE2) as a SARS-CoV-2 receptor: molecular mechanisms and potential therapeutic target. Intensive Care Med.

[26] Wrapp D, Wang N, Corbett KS, Goldsmith JA, Hsieh CL, Abiona O (2020). Cryo-EM structure of the 2019-nCoV spike in the prefusion conformation. Science.

[27] Herrera NG, Morano NC, Celikgil A, Georgiev GI, Malonis RJ, Lee JH (2020). Characterization of the SARS-CoV-2 S protein: biophysical, biochemical, structural, and antigenic analysis. BioRxiv. 2020.

[28] Li F (2016). Structure, function, and evolution of Coronavirus spike proteins. Annu Rev Virol.

[29] Padron-Regalado E (2020). Vaccines for SARS-CoV-2: lessons from other Coronavirus strains. Infect Dis Ther.

[30] McBride R, van Zyl M, Fielding BC (2014). The coronavirus nucleocapsid is a multifunctional protein. Viruses.

[31] Surjit M, Liu B, Chow VTK, Lal SK (2006). The nucleocapsid protein of severe acute respiratory syndrome-coronavirus inhibits the activity of cyclin-cyclin-dependent kinase complex and blocks S phase progression in mammalian cells. J Biol Chem.

[32] He R, Leeson A, Andonov A, Li Y, Bastien N, Cao J (2003). Activation of AP-1 signal transduction pathway by SARS coronavirus nucleocapsid protein. Biochem Biophys Res Commun.

[33] Satarker S, Nampoothiri M (2020). Structural proteins in Severe Acute Respiratory Syndrome Coronavirus-2. Arch Med Res.

[34] Yoshimoto FK (2020). The proteins of Severe Acute Respiratory Syndrome Coronavirus-2 (SARS CoV-2 or n-COV19), the cause of COVID-19. Protein J.

[35] Siu YL, Teoh KT, Lo J, Chan CM, Kien F, Escriou N (2008). The M, E, and N structural proteins of the severe acute respiratory syndrome coronavirus are required for efficient assembly, trafficking, and release of virus-like particles. J Virol.

[36] Tsoi H, Li L, Chen ZS, Lau K-F, Tsui SKW, Chan HYE (2014). The SARS-coronavirus membrane protein induces apoptosis via interfering with PDK1-PKB/Akt signalling. Biochem J.

[37] Verdiá-Báguena C, Nieto-Torres JL, Alcaraz A, DeDiego ML, Torres J, Aquilella VM, Enjuanes L (2012). Coronavirus E protein forms ion channels with functionally and structurally-involved membrane lipids. Virology.

[38] Nieto-Torres JL, DeDiego ML, Verdiá-Báguena C, Jimenez-Guardeño JM, Regla-Nava JA, Fernandez-Delgado R (2014). Severe acute respiratory syndrome coronavirus envelope protein ion channel activity promotes virus fitness and pathogenesis. PLoS Pathog.

[39] Chen W-H, Strych U, Hotez PJ, Bottazzi ME (2020). The SARS-CoV-2 vaccine pipeline: an overview. Curr Trop Med Rep.

[40] Singh K, Mehta S (2016). The clinical development process for a novel preventive vaccine: An overview. J Postgrad Med.

[41] Draft landscape of COVID-19 candidate vaccines. Available at:.

[42] Rajão DS, Pérez DR (2018). Universal vaccines and vaccine platforms to protect against influenza viruses in humans and agriculture. Front Microbiol.

[43] Soema PC, Kompier R, Amorij J-P, Kersten GFA (2015). Current and next generation influenza vaccines: formulation and production strategies. Eur J Pharm Biopharm.

[44] van Riel D, de Wit E (2020). Next-generation vaccine platforms for COVID-19. Nat Mater.

[45] Liu X, Liu C, Liu G, Luo W, Xia N (2020). COVID-19: Progress in diagnostics, therapy and vaccination. Theranostics.

[46] Wallis J, Shenton DP, Carlisle RC (2019). Novel approaches for the design, delivery and administration of vaccine technologies. Clin Exp Immunol.

[47] Wang J, Peng Y, Xu H, Cui Z, Williams RO (2020). The COVID-19 vaccine race: challenges and opportunities in vaccine formulation. AAPS PharmSciTech.

[48] Minor PD (2015). Live attenuated vaccines: historical successes and current challenges. Virology.

[49] Jiang S, Bottazzi ME, Du L, Lustigman S, Tseng CTK, Curti E (2012). Roadmap to developing a recombinant coronavirus S protein receptor-binding domain vaccine for severe acute respiratory syndrome. Expert Rev Vaccines.

[50] Romero-Maldonado A, Salazar-González JA, Rosales-Mendoza S (2014). Plant-based vaccines against influenza. In: Rosales-Mendoza S, Ed. Genetically engineered plants as a source of vaccines against wide spread diseases: an integrated view. New York, NY: Springer.

[51] Keech C, Albert G, Cho I, Robertson A, Reed P, Neal S (2020). Phase 1-2 trial of a SARS-CoV-2 recombinant spike protein nanoparticle vaccine. N Engl J Med.

[52] Noranate N, Takeda N, Chetanachan P, Sittisaman P, A-Nuegoonpipat A, Anantapreecha S (2014). Characterization of chikungunya virus-like particles. PloS One.

[53] Syomin BV, Ilyin YV (2019). Virus-like particles as an instrument of vaccine production. Mol Biol.

[54] Daniell H, Singh ND, Mason H, Streatfield SJ (2009). Plant-made vaccine antigens and biopharmaceuticals. Trends Plant Sci.

[55] Zeltins A (2013). Construction and characterization of virus-like particles: a review. Mol Biotechnol.

[56] Pillet S, Aubin É, Trépanier S, Bussiere D, Dargis M, Poulin JF (2016). A plant-derived quadrivalent virus like particle influenza vaccine induces cross-reactive antibody and T cell response in healthy adults. Clin Immunol Orlando Fla.

[57] Kaiser J (2008). Is the drought over for pharming?. Science.

[58] Navarre C, Delannoy M, Lefebvre B, Nader J, Vanham D, Boutry M (2006). Expression and secretion of recombinant outer-surface protein A from the Lyme disease agent, *Borrelia burgdorferi*, in *Nicotiana tabacum* suspension cells. Transgenic Res.

[59] Nelson G, Marconi P, Periolo O, La Torre J, Alvarez MA (2012). Immunocompetent truncated E2 glycoprotein of bovine viral diarrhea virus (BVDV) expressed in *Nicotiana tabacum* plants: a candidate antigen for new generation of veterinary vaccines. Vaccine.

[60] Łojewska E, Sakowicz T, Kowalczyk A, Konieczka M, Grzegorczyk J, Sitarek P (2020). Production of recombinant colicin M in *Nicotiana tabacum* plants and its antimicrobial activity. Plant Biotechnol Rep.

[61] Folegatti PM, Ewer KJ, Aley PK, Angus B, Becker S, Belij-Rammerstorfer S (2020). Safety and immunogenicity of the ChAdOx1 nCoV-19 vaccine against SARS-CoV-2: a preliminary report of a phase 1/2, single-blind, randomised controlled trial. Lancet.

[62] Zhu F-C, Li Y-H, Guan X-H, Hou LH, Wang WJ, Li JX (2020). Safety, tolerability, and immunogenicity of a recombinant adenovirus type-5 vectored COVID-19 vaccine: a dose-escalation, open-label, non-randomised, first-in-human trial. Lancet.

[63] Zhu F-C, Guan X-H, Li Y-H, Huang JY, Jiang T, Hou LH (2020). Immunogenicity and safety of a recombinant adenovirus type-5-vectored COVID-19 vaccine in healthy adults aged 18 years or older: a randomised, double-blind, placebo-controlled, phase 2 trial. Lancet.

[64] Logunov DY, Dolzhikova IV, Zubkova OV, Tukhvatullin AI, Shcheblyakov DV, Dzharullaeva AS (2020). Safety and immunogenicity of an rAd26 and rAd5 vector-based heterologous prime-boost COVID-19 vaccine in two formulations: two open, non-randomised phase 1/2 studies from Russia. Lancet.

[65] Pardi N, Hogan MJ, Porter FW, Weissman D (2018). mRNA vaccines - a new era in vaccinology. Nat Rev Drug Discov.

[66] Wang F, Kream RM, Stefano GB (2020). An evidence based perspective on mRNA-SARS-CoV-2 vaccine development. Med Sci Monit.

[67] Schlake T, Thess A, Fotin-Mleczek M, Kallen K-J (2012). Developing mRNA-vaccine technologies. RNA Biol.

[68] Corbett KS, Flynn B, Foulds KE, Francica JR, Boyoglu-Barnum S, Werner AP (2020). Evaluation of the mRNA-1273 vaccine against SARS-CoV-2 in nonhuman primates. N Engl J Med.

[69] Jackson LA, Anderson EJ, Rouphael NG, Roberts PC, Makhene M, Coler RN (2020). An mRNA vaccine against SARS-CoV-2 - preliminary report. N Engl J Med.

[70] Liu MA (2019). A comparison of plasmid DNA and mRNA as vaccine technologies. Vaccines.

[71] Choe PG, Perera R a (2017). PM, Park WB, Song KH, Bang JH, Kim ES, et al. MERS-CoV antibody responses 1 year after symptom onset, South Korea, 2015. Emerg Infect Dis.

[72] Wu L-P, Wang N-C, Chang Y-H, Tian XY, Na DY, Zhang LY (2007). Duration of antibody responses after Severe Acute Respiratory Syndrome. Emerg Infect Dis.

[73] Liu L, Wei Q, Lin Q, Fang J, Wang H, Kwok H (2019). Anti-spike IgG causes severe acute lung injury by skewing macrophage responses during acute SARS-CoV infection. JCI Insight.

[74] A vaccine for SARS-CoV-2: goals and promises. EClinicalMedicine. 2020; 24. Available at:.

[75] Sewell HF, Agius RM, Stewart M, Kendrick D (2020). Cellular immune responses to covid-19. BMJ.

[76] Wang X, Guo X, Xin Q, Pan Y, Hu Y, Li J (2020). Neutralizing antibody responses to Severe Acute Respiratory Syndrome Coronavirus 2 in Coronavirus disease 2019 inpatients and convalescent patients. Clin Infect Dis.

[77] Iwasaki A (2020). What reinfections mean for COVID-19. Lancet Infect Dis.

[78] Tillett RL, Sevinsky JR, Hartley PD, Kerwin H, Crawford N, Gorzalski A (2020). Genomic evidence for reinfection with SARS-CoV-2: a case study. Lancet Infect Dis.

[79] Lee WS, Wheatley AK, Kent SJ, DeKosky BJ (2020). Antibody-dependent enhancement and SARS-CoV-2 vaccines and therapies. Nat Microbiol.

[80] How and when will we know that a COVID-19 vaccine is safe and effective? Available at:.

[81] Phillips N, Cyranoski D, Mallapaty S (2020). A leading coronavirus vaccine trial is on hold: scientists react. Nature.

[82] Plans-Rubió P (2012). The vaccination coverage required to establish herd immunity against influenza viruses. Prev Med.

[83] Plans P, Torner N, Godoy P, Jané M (2014). Lack of herd immunity against measles in individuals aged <35 years could explain re-emergence of measles in Catalonia (Spain). Int J Infect Dis.

[84] Randolph HE, Barreiro LB (2020). Herd immunity: understanding COVID-19. Immunity.

[85] Raimondi MT, Donnaloja F, Barzaghini B, Bocconi A, Conci C, Parodi V (2020). Bioengineering tools to speed up the discovery and preclinical testing of vaccines for SARS-CoV-2 and therapeutic agents for COVID-19. Theranostics.

[86] Funk CD, Laferrière C, Ardakani A (2020). A Snapshot of the global race for vaccines targeting SARS-CoV-2 and the COVID-19 pandemic. Front Pharmacol.

[87] Langat P, Raghwani J, Dudas G, Bowden TA, Edwards S, Gall A (2017). Genome-wide evolutionary dynamics of influenza B viruses on a global scale. PLoS Pathog.

[88] Mercatelli D, Giorgi FM (2020). Geographic and genomic distribution of SARS-CoV-2 mutations. Front Microbiol.

[89] Shang W, Yang Y, Rao Y, Rao X (2020). The outbreak of SARS-CoV-2 pneumonia calls for viral vaccines. Npj Vaccines.

[90] Versteeg L, Almutairi MM, Hotez PJ, Pollet J (2019). Enlisting the mRNA vaccine platform to combat parasitic infections. Vaccines.

[91] Robbins G, Wosen J. Scientists are struggling to quickly find a vaccine that can vanquish coronavirus. San Diego Union-Tribune. 2020. Available at:.

